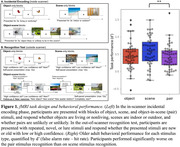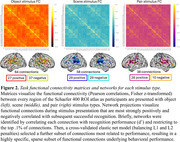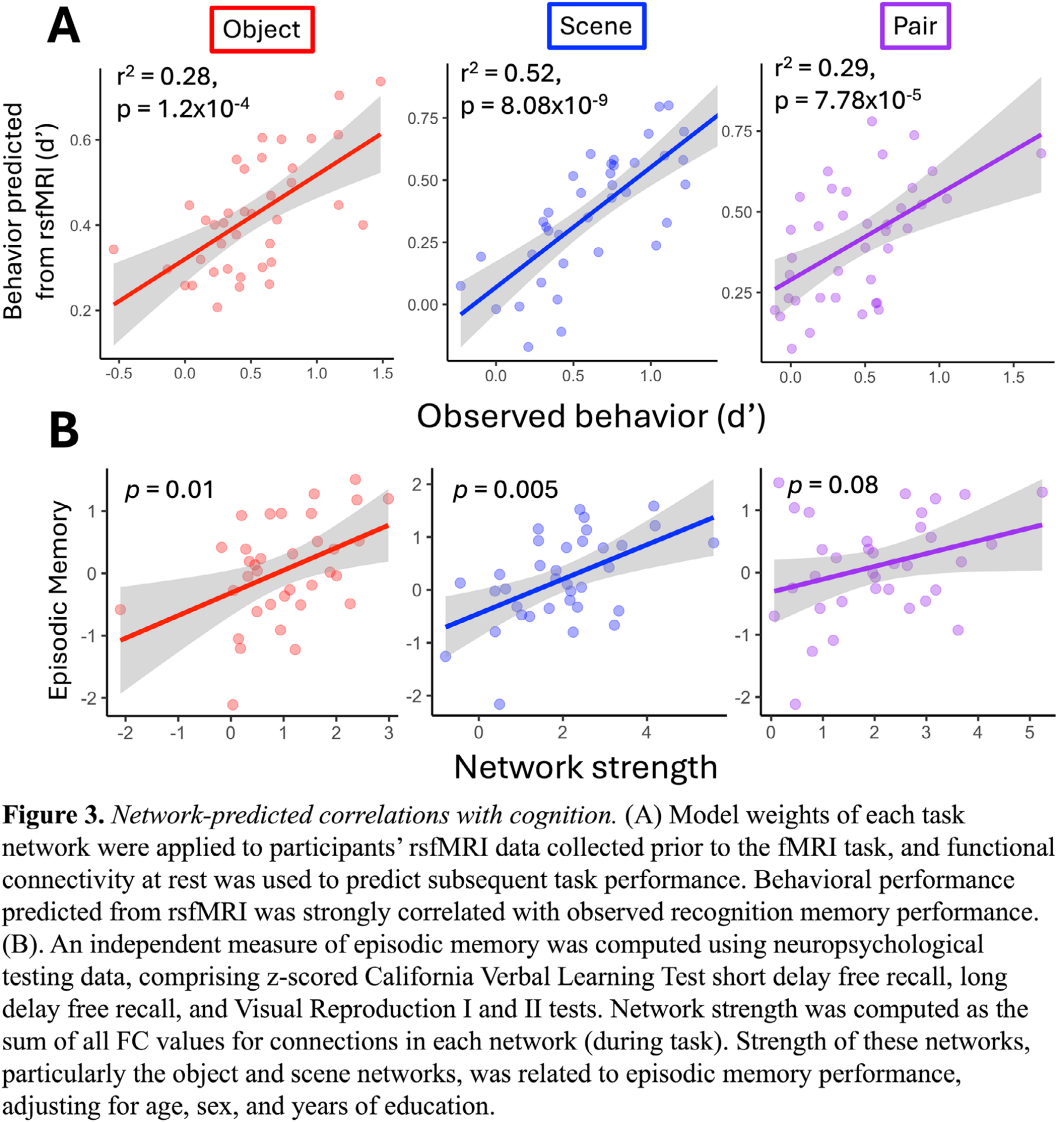# Task fMRI networks predict memory in cognitively unimpaired older adults

**DOI:** 10.1002/alz.092892

**Published:** 2025-01-09

**Authors:** Jacob Ziontz, Xi Chen, William J. Jagust

**Affiliations:** ^1^ University of California, Berkeley, Berkeley, CA USA; ^2^ Lawrence Berkeley National Laboratory, Berkeley, CA USA; ^3^ Stony Brook University, Stony Brook, NY USA

## Abstract

**Background:**

Episodic memory decline is a hallmark feature of aging and Alzheimer’s disease, but the mechanisms underlying its earliest stages are unknown.

**Method:**

Cognitively unimpaired older adults from the Berkeley Aging Cohort Study (n=49) completed an fMRI memory encoding task preceded by 15 minutes rsfMRI and standard neuropsychological testing. Participants viewed object, scene, and object‐in‐scene (pair) stimuli in the scanner for 40 minutes. They then completed a recognition test to distinguish repeated from novel stimuli, and their performance was quantified as d’ (false alarm rate – hit rate; Figure 1). Functional connectivity (FC) was computed between all regions of the Schaefer 400 ROI atlas during presentation of each stimulus type and during rest. An elastic net filter model approach identified a sparse subset of connections highly related to successful performance in each task component. Model weights for these connections were applied to each participant’s rsfMRI connectivity to predict performance on each task component. Network strength was computed by summing FC values for each connection in the network.

**Result:**

Task fMRI networks comprised unique sets of connections positively and negatively related to performance in each task component: the object network included 27 positive and 37 negative connections, the scene network included 29 positive and 29 negative connections, and the pair network include 28 positive and 10 negative connections (Figure 2). Applied to rsfMRI data collected prior to task, respective networks predicted object (r^2^ = 0.28, p < 0.001), scene (r^2^ = 0.52, p < 0.001), and pair (r^2^ = 0.29, p < 0.001) recognition performance (Figure 3a). Adjusting for age, sex, and education, strength of object (b = 0.36, p = 0.013) and scene networks (b = 0.33, p = 0.005) were correlated with a composite measure of episodic memory performance generated from a neuropsychological test battery (Figure 3b).

**Conclusion:**

Functional networks that support successful memory encoding in older adults can be applied to rsfMRI to predict performance, and the strength of these networks is further associated with independent measures of episodic memory. Future work will investigate the potential for these networks to predict the earliest stages of cognitive decline.